# Editorial: Expanding the reach of evidence-based psychological interventions for mental health: innovation, access, and equity

**DOI:** 10.3389/fpsyt.2026.1787162

**Published:** 2026-02-23

**Authors:** Tamara Melnik, Jorge Sinval, Fernanda Machado Lopes

**Affiliations:** 1 Paulista School of Medicine, Federal University of São Paulo, São Paulo, SP, Brazil; 2 National Institute of Education, Nanyang Technological University, Singapore, Singapore; 3 Faculty of Medicine, University of Lisbon, Lisbon, Portugal; 4 Business Research Unit (BRU-IUL), University Institute of Lisbon (Iscte-IUL), Lisbon, Portugal; 5 Faculty of Philosophy, Sciences and Letters at Ribeirão Preto, University of São Paulo, Ribeirão Preto, SP, Brazil; 6 Laboratory of Basic and Applied Cognitive Psychology (LPCog) [Laboratório de Psicologia Cognitiva Básica e Aplicada], Federal University of Santa Catarina (UFSC), Florianópolis, SC, Brazil

**Keywords:** evidence-based psychology, GRADE approach, implementation, meta-analysis, systematic review, evidence-based practice in psychology

## The evolution of evidence-based psychological practice

Evidence-Based Psychological Practice (EBPP) reached a new level of methodological and conceptual perspective in 2025. More than three decades after the American Psychological Association (APA) Division 12 introduced criteria for Empirically Supported Treatments (1) and following their refinement by Tolin et al. (2), EBPP has evolved beyond a narrow focus on experimental efficacy. Contemporary EBPP increasingly reflects an integrated evidence ecosystem that prioritizes not only internal validity, but also contextual relevance, equity, feasibility, and clinical utility across diverse populations and health systems.

A central element of this evolution has been the growing alignment between the APA model of EBPP and international frameworks for evidence appraisal, particularly the Grading of Recommendations Assessment, Development and Evaluation (GRADE) approach (3). While the APA framework emphasizes the integration of the best available research evidence with clinical expertise and patient characteristics, values (4), GRADE provides a transparent and systematic methodology for assessing the certainty of evidence and the strength of recommendations (5). Together, these frameworks have strengthened EBPP by shifting the field away from dichotomous judgments of efficacy toward a more explicit and nuanced consideration of certainty, trade-offs, contextual factors, and equity. This convergence has contributed to a more transparent, decision-oriented, internationally coherent model of evidence to inform psychological practice.

In Latin America, this evolution has been marked by a set of pioneering initiatives aimed at strengthening professional training, methodological literacy, and the structured implementation of evidence-based approaches in psychology. Among these initiatives, the publication of *Prática da Psicologia Baseada em Evidências* (6) represents a landmark contribution by systematically integrating international frameworks — such as those of the American Psychological Association (APA), the Cochrane Collaboration, and the Grading of Recommendations, Assessment, Development and Evaluation (GRADE) Working Group — into a comprehensive and context-sensitive framework for psychological practice in Portuguese-speaking countries. This work has played a central role in disseminating evidence-based principles, fostering skills in critical appraisal, and supporting transparent clinical and policy-oriented decision-making.

Complementing this conceptual and educational advance, empirical research has begun to document how EBPP is understood and applied in clinical settings. The cross-sectional study titled *Knowledge and Use of Evidence-Based Practice in Psychology in the Clinical Practice of Brazilian Psychologists* constitutes one of the first systematic efforts in Latin America to assess psychologists’ (7) knowledge, attitudes, and use of EBPP in everyday practice. Together, these initiatives signal an important transition from theoretical endorsement to empirical evaluation and the structured implementation of EBPP within the Brazilian context, contributing to the consolidation of a regional evidence-based culture aligned with global standards.

The Research Topic *“Expanding the Reach of Evidence-Based Psychological Interventions for Mental Health: Innovation, Access, and Equity”* exemplifies this transition. By bringing together systematic reviews, study protocols, meta-analyses, qualitative syntheses, and implementation studies, the Research Topic illustrates how EBPP is increasingly oriented toward translational relevance and practical, real-world applicability across diverse cultural and clinical contexts.

## Methodological evolution: from efficacy to certainty of evidence

One of the most salient methodological advances represented in this Special Issue is the growing emphasis on the certainty of evidence. The umbrella review protocol by Lerner et al. exemplifies this shift by proposing a meta-epidemiological assessment of the certainty of evidence in systematic reviews of cognitive-behavioral and psychodynamic/psychoanalytic therapies. Anchored in the GRADE framework, the protocol reflects the maturation of EBPP by explicitly addressing confidence in effect estimates, rather than focusing solely on whether interventions demonstrate statistically significant outcomes.

By applying a shared evaluative framework across distinct psychotherapeutic traditions, this work facilitates comparability and methodological dialogue between approaches that have historically been assessed in parallel. In doing so, it advances an approach that supports more informed clinical, educational, and policy decisions.

Complementing this contribution, the protocol by Yao et al. applies network meta-analysis to compare multiple electrical stimulation modalities for depression. By evaluating psychosocial and neurobiological interventions within a unified analytical framework, the study reinforces an integrated understanding of mind and behavior and illustrates how diverse intervention models can be appraised using consistent methodological standards. This integration clarifies the relative efficacy and safety of these modalities and enhances the coherence of evidence synthesis in mental health.

## Integration of psychotherapeutic and pharmacological approaches

The systematic review by Santos et al. on pharmacological and psychosocial interventions for night eating syndrome in adults exemplifies the biopsychosocial orientation of contemporary EBPP. Rather than positioning medications and psychological interventions as competing alternatives, the review frames them as complementary components of a therapeutic continuum that is appropriate for addressing complex, multifactorial conditions.

By synthesizing evidence across multiple outcomes, including symptom severity, weight-related measures, quality of life, and comorbidities, the study contributes to the organization of an emerging but clinically relevant field. While highlighting the potential benefits of interventions such as sertraline and progressive muscle relaxation, the review also identifies substantial gaps in the evidence base and underscores the need for more robust trials to inform practice and guideline development.

In the domain of psychosocial interventions, Huang et al. provide a comprehensive comparative synthesis of international clinical practice guidelines for anxiety disorders in adults. By identifying areas of convergence, such as the consistent recommendation of cognitive-behavioral therapy, alongside critical gaps, including the lack of guidance for separation anxiety disorder in adults, the review has direct implications for clinical practice. The rigorous appraisal of guideline quality using Appraisal of Guidelines for Research and Evaluation further establishes methodological benchmarks for future guideline development and evaluation.

## Interactions between individual and contextual mechanisms

Several contributions in this Research Topic address the interaction between individual mechanisms and broader contextual factors, reflecting the EBPP emphasis on contextualization and shared decision-making. Lozano et al. examine how implementation climate influences primary care professionals’ attitudes toward preventive mental health and substance use intervention for Hispanic families. Using multilevel modeling, the study demonstrates that favorable individual perceptions of implementation climate do not automatically translate to the clinic level, highlighting organizational heterogeneity and the need for context-sensitive implementation strategies, particularly in populations affected by health inequities.

Hall et al. contributes a qualitative meta-synthesis of dialectical behavior therapy (DBT), focusing on patients’ subjective experiences across formats and clinical settings. Emotional validation, therapeutic alliance, and sense of agency emerge as central mechanisms of change, underscoring the critical role of qualitative evidence in understanding therapeutic effectiveness. By illuminating processes that often escape traditional quantitative metrics, the synthesis strengthens the conceptual foundations of EBPP and supports more flexible, patient-centered models of care.

Zheng et al. further advance mechanism-focused research by demonstrating that mindfulness reduces work addiction among young employees through increased cognitive reappraisal and reduced maladaptive perfectionism. These findings suggest that mindfulness-based organizational interventions may enhance emotional regulation and promote a healthier work–life balance, with implications for prevention and occupational mental health.

## Digital mental health interventions

Digital interventions represent a rapidly expanding frontier for EBPP. Hall et al. offer a practice-oriented analysis of the commissioning of digital mental health interventions within the UK National Health Service. Using the Online Remote Behavioral Intervention for Tics (ORBIT) as a case study, the authors identify structural barriers, such as unclear commissioning pathways and challenges related to integration, alongside facilitators, including strong evidence bases and clinical advocacy. This work provides actionable insights for bridging innovation, evidence, and routine clinical care.

Finally, Wen et al. present a meta-analysis examining treatment discontinuation in remotely delivered cognitive remediation interventions for individuals with schizophrenia. By comparing dropout rates across remote and in-person formats and identifying factors associated with better adherence, the study offers practical guidance for optimizing digital intervention design. These findings are particularly relevant for expanding access to effective, equitable, and user-centered mental health services.

## Concluding remarks: toward a global evidence ecosystem

Collectively, the studies in this Special Issue illustrate the ongoing transformation of Evidence-Based Psychological Practice into a global and integrated evidence ecosystem. By foregrounding certainty of evidence, contextual relevance, implementation processes, and patient-centered outcomes, EBPP is increasingly aligned with principles of transparency, equity, and real-world applicability. The convergence of the APA and GRADE frameworks, alongside advances in qualitative synthesis, implementation science, and digital mental health, signals a future in which psychological practice is guided not only by what works, but by how, for whom, and under what conditions interventions are most effective.

This evolving model positions EBPP as a cornerstone of contemporary mental health care, capable of informing clinical decision-making, guideline development, and mental health policy in an increasingly complex and diverse global landscape.
